# Adherence to Typical Antipsychotics among Patients with Schizophrenia in Uganda: A Cross-Sectional Study

**DOI:** 10.1155/2023/7035893

**Published:** 2023-02-03

**Authors:** Moses Kule, Mark Mohan Kaggwa

**Affiliations:** ^1^Department of Psychiatry, Mbarara Regional Referral Hospital, Mbarara, Uganda; ^2^Department of Psychiatry and Behavioural Neurosciences, McMaster University, Canada; ^3^Department of Psychiatry, Mbarara University of Science and Technology, Uganda

## Abstract

**Background:**

There has been a recent transition from typical to atypical antipsychotics in managing schizophrenia. This has been attributed to the acute side effects experienced by patients on typical antipsychotics that lead to nonadherence. However, the treatment cost with typical antipsychotics is cheaper (preferred in low-income settings), and there is no difference in the effectiveness, efficacy, discontinuation rate, or side effect symptom burden with atypical antipsychotics. This study is aimed at determining the prevalence of nonadherence and the associated factors to typical antipsychotics among patients with schizophrenia attending a psychiatric outpatient clinic at a rural tertiary facility in Uganda.

**Method:**

A cross-sectional study among 135 patients with schizophrenia for at least six months on typical antipsychotics (mean age of 39.7 (±11.9) and 55.6% were female) from a rural tertiary facility in Uganda. Data were collected regarding sociodemographics, adherence, insight for psychosis, attitude towards typical antipsychotics, side effects, satisfaction with medications, and explanations from health workers about medications and side effects. Logistic regression was used to determine the factors associated with nonadherence.

**Results:**

The prevalence of nonadherence was 16.3%, and the likelihood of being nonadherent was more among the poor (monthly earning below the poverty line). However, having reduced energy was associated with reducing the likelihood of having nonadherence.

**Conclusion:**

The prevalence of nonadherence was lower than many previously obtained prevalence and was comparable to nonadherence for atypical antipsychotics. However, to reduce nonadherence, we need all stakeholders (such as the government, insurance companies, and caregivers) to assist patients living in poverty with access to medication.

## 1. Background

Schizophrenia is a multidimensional disorder with several subtypes, different neurobiological underpinnings, and several lines of treatment to control the symptoms—mainly antipsychotics [1–3]. However, over the past years, a lack of adherence to antipsychotic drugs has emerged as one of the clinicians' biggest challenges in treating and managing schizophrenia patients [4, 5]. Antipsychotic drugs have been the mainstay in treating schizophrenia and have shown effectiveness in reducing the severity of psychotic symptoms and preventing relapses in the first year of acute psychotic episodes [6, 7]. Antipsychotics are classified into two major classes, i.e., typical (e.g., haloperidol and chlorpromazine) and atypical (e.g., olanzapine and risperidone). Recently, the proportion of individuals receiving atypical antipsychotics has increased [8, 9]. However, based on a randomized controlled trial meta-analysis among individuals with first-episode psychosis, there were no reported differences between typical and atypical antipsychotics on the effectiveness, efficacy, discontinuation rate, and side effect symptom burden [10]. Interestingly, typical antipsychotics, especially haloperidol, prevented relapses better than most atypical antipsychotics [11]. Based on recent network meta-analyses published in *The Lancet* about the comparative efficacy and tolerability of antipsychotics, no clear difference exists between most antipsychotics for relapse prevention and efficacy; but the differences were marked in side effects profile and tolerability [12, 13]. Nonadherence to typical antipsychotics has remained a bigger challenge than atypical antipsychotic drugs [14]. This is mainly attributed to the multiple acute intolerable side effects experienced (e.g., extrapyramidal side effects (EPS)) while using the typical antipsychotic [14–17]. The acute side effects, especially EPS, can be controlled and managed using anticholinergics and other modalities to reduce patient burden [4]. Typical antipsychotics are also associated with fewer metabolic diseases making long-term care in complication management cheaper. Furthermore, the treatment cost while using typical antipsychotics is statistically more affordable than atypical antipsychotics [18].

Apart from side effects, other factors associated with nonadherence to typical antipsychotics include the following: negative attitude towards the medications, comorbid medical and psychiatric conditions such as depression, polytherapy, poor insight for psychotic symptoms, irritability, use of substances of addiction (e.g., *khat* and alcohol), the severity of the psychotic symptoms, lack of social support, cognitive impairment, and inadequate therapeutic alliance [4, 16, 17, 19, 20]. In addition, younger patients (below 30 years), those with lower levels of education, having a longer duration of illness (above five years), and those living in extreme poverty have greater levels of nonadherence [4, 16, 21, 22].

Nonadherence leads to frequent psychiatric hospitalization, use of emergency psychiatric services, arrests, violence, victimizations, poorer mental functioning, suicidality, poorer life satisfaction, greater substance use and related problems, and persistent reoccurrence of psychotic symptoms in patients, which lead to increasing the health care costs and resource utilization that increases patient and society financial and economic needs [4, 23–28].

The prevalence of nonadherence to typical antipsychotics in Africa is relatively higher than other continents, i.e., ranges between 46.9% and 93.3% in Africa [17, 20, 29–31] vs. between 11% in China and 25.6% in Bulgaria [19, 22, 32, 33] in other continents. With all the consequences and factors associated with nonadherence, little is still known about the adherence to typical antipsychotic medications among patients with schizophrenia in Uganda. Therefore, this study determined the prevalence of nonadherence to typical antipsychotic medications and associated factors among patients with schizophrenia at a rural psychiatric facility in Uganda. In Uganda, mental health care is transitioning to the use of atypical antipsychotics, as seen in other parts of the world [8, 9], and typically is more common in rural settings.

## 2. Methods

### 2.1. Study Design, Area, and Population

A cross-sectional study was conducted between January and July 2019 among patients diagnosed with schizophrenia based on the diagnostic and statistical manual five (DSM 5). Patients were on typical antipsychotics at a tertiary hospital psychiatric outpatient clinic in rural Uganda. The study included patients aged 18 and above who had been on treatment for at least six months. Patients that were psychologically or physically too ill to consent were excluded from the study. In addition, patients on (i) both typical and atypical antipsychotics, (ii) depot typical antipsychotics, and (iii) who had used typical antipsychotics for less than six months were also excluded. The total population of patients with schizophrenia on typical antipsychotics attending the clinic was 150 as of November 2018 (before the commencement of the study) based on the tertiary facility hospital records. The included participants were recruited consecutively.

### 2.2. Data Collection Procedure and Study Measures

After patients were reviewed during their outpatient clinic visit, the research assistant (a psychiatry nurse) approached the eligible participants and explained the study's objective. After obtaining informed consent, participants were given a questionnaire in their preferred language that captured the following information: (i) sociodemographics, (ii) insight for psychosis, (iii) attitude towards typical antipsychotics, (iv) side effects of typical antipsychotics, (v) satisfaction with typical antipsychotics, and (vi) adherence to the typical antipsychotics.

#### 2.2.1. Sociodemographics

Information captured included age, gender, level of education (none, primary, secondary, or tertiary), area of residence, marital status, income status (below vs. above countries poverty line per month, i.e., 16643 per person per month in Uganda shillings [34, 35]), distance from the hospital in kilometers, use of substance abuse, and presence of a comorbidity.

#### 2.2.2. The Insight Scale for Psychosis (ISP)

The 8-item self-report ISP was used to assess insight into psychosis among patients with schizophrenia [36]. The responses are rated on a three-point scale from 0 (disagree) to 2 (agree), and the total score ranges between 0 and 16, where a higher score indicates more insight, and scores of nine and above imply good insight [36]. However, we rephrased one item: “My stay in the hospital is necessary.” This item was substituted with “My return for reviews is necessary” since we enrolled only outpatients. The eight items are organized into three subscales, i.e., awareness of illness (items 1 and 8), relabeling of symptoms (items 2 and 7), and need for treatment (items 3, 4, 5, and 6) [36, 37].

#### 2.2.3. Drug Attitude Inventory (DAI)

The DAI assessed patients' attitudes and beliefs towards typical antipsychotic medication. The tool was derived from the Hogan's Drug Attitude Inventory of 30 items [38] and remodified to a 10-item questionnaire; these scales have similar reliability [39]. DAI is a 10-item scale with *Yes*/*No* responses to the questions. A correct response on an item was scored as +1, meaning a positive attitude, and an incorrect −1, meaning a negative attitude. The final score was a sum of the scores on the responses. A positive total score indicates a positive attitude, while a negative total score indicates a negative attitude towards typical antipsychotic medication. However, patients receiving a total of 0 were considered neutral.

#### 2.2.4. The Modified Version of the Medication Adherence Rating Scale (MARS)

The MARS was used to assess medication adherence among the study participants. MARS is a 10-item self-report tool developed by Thompson et al. with a multisection, i.e., medication adherence behavior (items 1-4), attitude towards taking medication (items 5-8), and adverse side effects and attitudes to psychotropic medication (items 9-10) [40]. The items are answerable by a *Yes/No* response, scored as one and zero, respectively. Some questions required a reverse score, i.e., 1-6 and 9-10; here, a no response was scored one [40]. The total scores ranged between 0 and 10, with a higher score indicating better medication adherence [40]. A cutoff of three was used for adherence (i.e., two and below were for nonadherence). The tool has been used in a similar setting (Nigeria), and it had good reliability (Cronbach's alpha of 0.76) [41].

#### 2.2.5. Antipsychotic Side Effect Checklist (ASC)

The ASC-Clinic (ASC-C) clinician version was used to assess side effects experienced by patients during the clinical visit. It includes common side effects faced by patients taking typical antipsychotic medications and includes extrapyramidal side effects (EPS) and non-EPS-related questions [42]. Patients report the tool's importance in identifying their symptoms and improving their communication with clinicians. For example, in a multicenter pilot study, about 86% of patients reported that the tool helped them easily communicate with the psychiatrist [43]. A set of 17 semistructured questions was administered to the patient in the form of an interview, requiring a *Yes*/*No* response; a “No” response represented the absence of the side effect coded 0, while a “Yes” answer represented the presence of the side effect coded 1. A higher total score indicated more side effects experienced.

#### 2.2.6. Satisfaction with Medication and Explanation from Health Workers

One question adopted from a study by Magura et al. was used to assess satisfaction with the use of typical antipsychotics [44], i.e., “How satisfied are you with the psychiatric medications you have been taking?”. The responses ranged from 0 (not at all satisfied) to 5 (very satisfied). Based on a similar rating scale, patients were asked about their satisfaction with health works' explanations of the typical antipsychotic use and side effects.

#### 2.2.7. The Complexity of Regimens and Pill Burden

Two questions were asked: (i) “How often do you take your medication daily?” and (ii) “What is the total number of pills you take daily” to assess the complexity of the regimen and pill burden, respectively.

### 2.3. Ethical Considerations

The study was conducted in accordance with the Declaration of Helsinki 2013 [45]. The study received ethical approval from the research ethics committee of the institution. All participants provided voluntary written informed consent to participate in the study.

### 2.4. Statistical Analysis

Data were entered into *Microsoft Excel* version 2010, cleaned, coded, and exported to STATA version 16 for analysis. Percentages and frequencies were reported for all the categorical variables, while mean and standard deviation were reported for continuous variables. A continuous variable was considered normally distributed if *kurtosis* was below seven and the *skewness* was below 2 [46]; otherwise, it was categorized. Pearson's correlation was used for the total scores of the different items to determine the relationship between adherence, insight into psychosis, attitude towards typical antipsychotics, and satisfaction with medications. However, the chi-square test was used to determine the relationship between adherence and side effects experienced. Logistic regression was used to identify the factors associated with nonadherence. Factors with a *p* value less than 0.3 at bivariate analysis were tested for collinearity. The factors with a VIF below three were considered for multivariate logistic regression analysis. A *p* value of less than 0.05 was considered statistically significant at a 95% confidence interval.

## 3. Results

### 3.1. Sociodemographic Characteristics

One hundred thirty-five patients diagnosed with schizophrenia on typical antipsychotics were recruited for the study. More than half (55.6%) were females; the majority, 85.2%, were Christians, 57.8% were staying in rural areas, and 37.0% were married. The mean age was 39.7 (±11.9). Patients with a secondary level of education were statistically significantly more likely to be nonadherent, followed by primary, then tertiary, and all noneducated adhered (23.9%, 20.4%, 3.0%, and 0, respectively, *p* = 0.042) (Table 1).

### 3.2. Relationship between Insight for Psychosis, Adherence, Attitude, and Satisfaction with Typical Antipsychotic Medications among Patients with Schizophrenia

Most of the patients (83.7% (*n* = 113)) were adhering to their treatment, with only 16.3% (*n* = 22) being nonadherent. Most clients (71.5%) had a positive attitude towards typical antipsychotic medication, and 74.8% had a good insight into psychosis. The mean satisfaction with typical antipsychotics was 3.7 (±1.0). Based on the continuous total scores on adherence, insight for psychosis, attitude towards drugs, and satisfaction with medication, we performed Pearson correlations to determine the relationship between the variables. There was a statistically significant positive correlation between the following pairs: (i) attitude towards medications and satisfaction with typical antipsychotics (*r*^2^ = 0.33) and (ii) satisfaction with medications taken and explanation from the health workers (*r*^2^ = 0.40). However, negative statistically significant correlations were between the following pairs: (i) adherence and attitude towards typical antipsychotics (*r*^2^ = −0.35), (ii) adherence and satisfaction with typical antipsychotics used (*r*^2^ = −0.30), and (iii) satisfaction with typical antipsychotics used and insight for psychosis (*r*^2^ = −0.19) (Table 2).

### 3.3. Treatment-Related Factors

The mean duration of treatment in years was 6.81 (SD = 7.24), a median of 4 years. Only 43 (31.9%) patients reported stopping medication for religious or cultural reasons. About half of the patients took medications daily (46.7%, *n* = 63); for details, see Table 3.

### 3.4. Relationship between Adherence Status and Side Effects Experienced

The most commonly experienced side effect was weight change (74.8%, *n* = 101/135) with the majority reporting an increase in weight (57.0%, 77/135) and 17.8% (n =24/135) reporting a decrease in weight. The second most experienced side effect was losing energy or drive, 65.9% (*n* = 89/135). The least experienced side effect was drooling of saliva, 14.8% (*n* = 20/135). Low energy or drive was statistically significantly greater among patients adhering to typical antipsychotics than those with nonadherence (91.0 vs. 9.0, *p* = 0.001) (Table 4).

### 3.5. Factors Associated with Nonadherence to Typical Antipsychotic Medications among Patients with Schizophrenia

The factors with a *p* value less than three were tested for collinearity, and the only number of side effects of typical antipsychotics had a VIF of above three (3.95). All remaining factors had a VIF of less than three with a mean VIF of 1.29. At multivariate logistic regression monthly income below poverty line was associated with being nonadherent (adjusted odds ratio = 7.15, 95% confidence interval: 1.39–36.67, *p* = 0.018). However, the likelihood of being nonadherent was reduced by 85% if an individual had a loss of energy as a side effect of the medications (i.e., if an individual experienced a loss of energy, they were more likely to adhere to their medication); for details, see Table 5. The final model had a goodness of fit *p* value of 0.733 for the 14 included variables. The model could correctly classify nonadherence of 86.2%, specificity of 96.9%, sensitivity of 35.0%, a positive predictive value of 70.0%, and a negative predictive value of 87.7%.

## 4. Discussion

The present study determined the prevalence and factors associated with nonadherence to typical antipsychotics among patients with schizophrenia in Uganda. The prevalence of nonadherence was 16.3%, and the likelihood of being nonadherent was higher among the poor; however, having reduced energy was associated with reducing the likelihood of nonadherence.

The prevalence of nonadherence in this study was lower than previously obtained in Africa, ranging between 46.9% and 93.3% [17, 20, 29–31]. The difference may be due to reduced stigma towards schizophrenia and improved mental health attitudes over the years [47]. In addition, the number of mental health care providers has increased. Thus, better services to provide care for those experiencing side effects and engaging more patients in adherence counseling and monitoring increased. The prevalence in the present study was similar to the prevalence of nonadherence to typical antipsychotics among high-income countries such as Germany (17.4%) [33] and Bulgaria [32]. Despite the similarities, these countries have very few individuals on typical antipsychotics, for example, only 23 participants in the German study [33]. Thus, adequate monitoring and screening for side effects reduces the likelihood of nonadherence. Also, with the many available options, such as atypical antipsychotics, the types are only given to individuals who have either failed the second generation or experienced adverse reactions [9]. For psychiatry service providers in low-income settings, evidence-based methods to enhance adherence, such as predischarge educational sessions, psychotherapeutic interventions, and telephone prompts, should be emphasized to help more patients adhere to typical antipsychotics [48, 49].

In this study, having a monthly income below the poverty line (i.e., 16643 per person per month in Uganda shillings) was associated with being nonadherent. The nonadherence is attributed to financial constraints that prevent them from buying the medication, as reported by other researchers in China [21, 22]. In addition, the medicines are expensive for the patients who are living with schizophrenia (majority unemployed), and being poor also makes them unable to afford transport to obtain the medication where they may be provided for free such as in some government-funded hospitals [50–54]. The poverty among patients with schizophrenia that leads to nonadherence was explored due to lack of access to government financial support, low awareness about government services, and little skills and training for certain jobs [53]. Furthermore, these patients cannot afford expensive insurance coverage, and few insurance companies cover mental health services. Therefore, to improve adherence, we recommend extensive health insurance coverage, government financial support, providing free or subsided cost medication, and financial incentives for schizophrenia patients, as suggested by other researchers [55–57].

In the present study, most patients experienced a loss of energy or drive (65.9%). The loss of energy or drive was more among patients adhering to typical antipsychotics than those with nonadherence. This finding was similar to an online direct-to-consumer questionnaire completed by 832 users of antipsychotics from 30 countries [58]. Studies have found that side effects are associated with medication nonadherence [14–17]. However, individuals with loss of energy or drive as a side effect of the medications were less likely to be nonadherent in the present study. This may be due to patients who are nonadherent having stopped taking the medication (i.e., becoming nonadherent) and not experiencing the side effect. However, this cannot be supported by the study design, and we propose a longitudinal study to identify the link between nonadherence and loss of energy and drive.

### 4.1. Limitations and Future Research

The present study has several limitations. First, a cross-sectional study precludes the establishment of causality among the variables. A longitudinal study could enlighten the causality. Second, the study had a small sample size that could not allow modeling testing, such as SEM, to show the relationship between the study variables and adherence clearly. Therefore, we recommend a multicenter study with a large sample size to enable further analysis and modeling. Third, the study was in one center, which precludes generalization of the findings to all patients living with schizophrenia on typical antipsychotics in Uganda. Four, the tools used have never been validated for use in Uganda. A study to validate some of these tools is recommended. Fifth, despite the various brands (market available brands) and types of typical antipsychotics (long acting vs. short acting) prescribed, we did not specify the type of typical antipsychotics or medication, which made conclusions about the individual medications difficult. In addition, the other medications patients were taking were also not specified; thus, we could not narrow down on how this interaction or their presence affects adherence. Six, selection bias was present in this study since we selected patients who had a diagnosis for over six months; these individuals would be used to the medications (developed tolerance) and experience fewer side effects, hence better adherence. Also, we may have selected individuals who had intellectual disabilities (ID) and are at a high likelihood of having poor adherence. We recommend future studies to adequately screen individuals with ID using reliable methods before excluding them or studying adherence among this unique population among individuals living with schizophrenia. Lastly, since the study asked individuals to remember aspects related to the use of typical antipsychotics, we may have had recall bias. To reduce recall bias and unreliability of study findings, future studies should incorporate both clinician-administered and self-report tools in understanding adherence phenomena among individuals living with schizophrenia.

## 5. Conclusion

The prevalence of nonadherence to typical antipsychotics was lower than the prevalence in previously obtained similar studies and was comparable to nonadherence for atypical antipsychotics. However, to reduce nonadherence, we need all stakeholders (such as the government, insurance companies, and caregivers) to assist patients living in poverty with access to medication.

## Figures and Tables

**Table 1:** Social demographic characteristics of patients with schizophrenia on typical antipsychotics.

Variable	*n* (%)	Adherence, 113 (83.7)	Nonadherence, 22 (16.3)	*p* value
Age (mean, SD)	39.7 (11.9)	39.9 (±11.4)	38.9 (±14.6)	0.727
Sex				
Male	60 (44.4)	53 (84.1)	10 (15.9)	0.901
Female	75 (55.6)	60 (83.3)	12 (16.7)	
Marital status				
Single	37 (27.4)	31 (83.8)	6 (16.2)	0.900
Married/cohabiting	50 (37.0)	41 (82.0)	9 (18.0)	
Separated/divorced	48 (35.6)	41 (85.4)	7 (14.6)	
Level of education				
None	7 (5.2)	7 (100)	0	0.042
Primary	49 (36.3)	39 (79.6)	10 (20.4)	
Secondary	46 (34.1)	35 (76.1)	11 (23.9)	
Tertiary	33 (24.4)	32 (97.0)	1 (3.0)	
Area residence				
Rural	78 (57.8)	65 (83.3)	13 (16.7)	0.892
Urban	57 (42.2)	48 (84.2)	9 (15.8)	
Poverty line				
Above	74 (54.8)	65 (87.8)	9 (12.2)	0.152
Below	61 (45.2)	48 (78.7)	13 (21.3)	
Substance use				
No	102 (75.6)	84 (82.3)	18 (17.7)	0.455
Yes	33 (24.4)	29 (87.9)	4 (12.1)	
Distance from the hospital in kilometers (mean, SD)				
Presence of a comorbidity	22.5 (±23.4)	21.0 (±22.2)	29.9 (±28.1)	0.103
No	97 (78.9)	83 (84.6)	14 (14.4)	0.289
Yes	26 (21.1)	20 (76.9)	6 (23.1)	

**Table 2:** Level of adherence to antipsychotic medications among schizophrenia patients.

Variable	*n* (%)	Adherence, 113 (83.7)	Nonadherence, 22 (16.3)	*p* value	Correlation between chosen study variables				
					1	2	3	4	5
Adherence (1)									
Adherence	113 (83.7)	113 (100)	0	<0.001	1				
Nonadherence	22 (16.3)	0	22 (100)						
Insight for psychosis (2)									
Good	101 (74.8)	84 (83.2)	17 (16.8)	0.772	0.02	1			
Poor	34 (25.2)	29 (85.3)	5 (14.7)						
Attitude towards typical antipsychotics (3)									
Negative	38 (28.2)	33 (86.8)	5 (13.2)	0.537	-0.35^∗^	-0.13	1		
Positive	97 (71.5)	80 (82.5)	17 (15.5)						
Satisfaction with medication (mean, SD) (4)	3.7 (±1.0)	3.6 (±1.0)	3.9 (±1.1)	0.381	-0.30^∗^	-0.19^∗^	0.33^∗^	1	
Satisfaction about health workers' explanation of typical antipsychotics use and side effects (5)	2.5 (±1.3)	2.6 (±1.3)	2.4 (±1.4)	0.597	-0.15	-0.04	0.15	0.40^∗^	1

**Table 3:** Treatment-related factors for patients with schizophrenia on typical antipsychotics.

Variable	*n* (%)	Adherence, 113 (83.7%)	Nonadherence, 22 (16.3%)	*p* value
Level of social support from the family and friends (mean, SD)	3.6 (±1.0)	3.7 (±1.0)	3.5 (±1.2)	0.562
Duration of treatment in years (mean, SD)	6.81 (±7.24)	7.0 (±7.4)	5.7 (±6.9)	0.457
Burdened by the number of pills				
No	88 (65.2)	74 (84.1)	14 (15.9)	0.868
Yes	47 (34.8)	39 (83.0)	8 (17.0)	
Number of pills (mean, SD)	3.44 (21.4)	3.6 (±2.1)	2.7 (±1.8)	0.089
Frequency of daily drug taking				
Once	63 (46.7)	50 (79.4)	13 (20.6)	0.254
Twice	55 (40.7)	48 (87.3)	7 (12.7)	
Three times	15 (11.1)	14 (93.3)	1 (6.7)	
Four times or more	2 (1.5)	1 (50.0)	1 (50.0)	
Frequency of drug stoke out at the facility for patients' medications				
Never	10 (7.4)	9 (90.0)	1 (10.0)	0.876
Rarely	21 (15.6)	17 (80.9)	4 (19.1)	
Sometimes	65 (48.1)	55 (84.6)	10 (15.4)	
Often	22 (16.3)	19 (86.4)	3 (13.6)	
Always	17 (12.6)	13 (76.5)	4 (23.5)	
Stop medications due to religious or cultural beliefs				
No	92 (68.1)	78 (84.8)	14 (15.2)	0.620
Yes	43 (31.9)	35 (81.4)	8 (18.6)	

**Table 4:** Relationship between adherence status and side effects experienced.

Side effect experienced	*n* (%)	Adherence, 113 (83.7)	Nonadherence, 22 (16.3)	*p* value
Loss of energy drive				
No	46 (34.1)	32 (69.6)	14 (30.4)	0.001
Yes	89 (65.9)	81 (91.0)	8 (9.0)	
Feeling unmotivated				
No	68 (50.4)	53 (77.9)	15 (22.1)	0.068
Yes	67 (49.6)	60 (89.5)	7 (10.5)	
Day time sedation				
No	59 (43.7)	47 (79.7)	12 (20.3)	0.262
Yes	76 (56.3)	66 (86.8)	10 (12.2)	
Too much sleep				
No	75 (55.6)	62 (82.7)	13 (17.3)	0.715
Yes	60 (44.4)	51 (85.0)	9 (15.0)	
Muscle too stiff				
No	100 (74.1)	85 (85.0)	15 (15.0)	0.491
Yes	35 (25.9)	28 (80.0)	7 (20.0)	
Shaking				
No	86 (63.7)	70 (81.4)	16 (18.6)	0.336
Yes	49 (36.3)	43 (87.8)	6 (12.2)	
Jittery				
No	94 (69.6)	77 (81.9)	17 (18.1)	0.394
Yes	41 (30.4)	36 (87.8)	5 (12.2)	
Akathisia				
No	104 (77.0)	85 (81.7)	19 (18.3)	0.256
Yes	31 (23.0)	28 (90.3)	3 (9.7)	
Trouble waking up				
No	66 (48.9)	55 (83.3)	11 (16.7)	0.909
Yes	69 (51.1)	58 (84.1)	11 (15.9)	
Vision changes				
No	96 (71.1)	78 (81.2)	18 (18.8)	0.226
Yes	39 (28.9)	35 (89.7)	4 (10.3)	
Dry mouth				
No	89 (65.9)	74 (83.1)	15 (16.9)	0.807
Yes	46 (34.1)	39 (84.8)	7 (15.2)	
Drooling of saliva				
No	115 (85.2)	96 (83.5)	19 (16.5)	0.865
Yes	20 (14.8)	17 (85.0)	3 (15.0)	
Memory problems				
No	72 (53.3)	60 (83.3)	12 (16.7)	0.901
Yes	63 (46.7)	53 (84.1)	10 (15.9)	
Constipation				
No	99 (73.3)	85 (85.9)	14 (14.1)	0.261
Yes	36 (26.7)	28 (77.8)	8 (22.2)	
Weight changes				
Decrease	24 (17.8)	22 (91.7)	2 (8.3)	0.294
Increase	77 (57.0)	65 (84.4)	12 (15.6)	
No change	34 (25.2)	26 (76.5)	8 (23.53)	
Sexual arousal				
No	63 (46.7)	53 (84.1)	10 (15.9)	0.901
Yes	72 (53.3)	60 (83.3)	12 (16.7)	
Menstrual changes				
No	113 (83.7)	97 (85.8)	16 (14.2)	0.128
Yes	22 (16.3)	16 (72.7)	6 (27.3)	
Total number of side effects experienced (mean, SD)	6.8 (±3.0)	7.0 (±2.9)	5.9 (±3.3)	0.136

**Table 5:** Factors associated with nonadherence to typical antipsychotic medications among patients with schizophrenia.

Variable	Bivariate logistic regression		Multivariate logistic regression	
	Crude odds ratio (95% confidence interval)	*p* value	Adjusted odds ratio (95% confidence interval)	*p* value
Age	0.99 (0.95–1.03)	0.724		
Sex				
Female	1			
Male	0.67 (0.26–1.72)	0.406		
Marital status				
Single	1			
Married/cohabiting	1.13 (0.36–3.52)	0.828		
Separated/divorced	0.88 (0.27–2.89)	0.836		
Level of education				
None	Omitted		Omitted	
Primary	8.21 (1.00–67.55)	0.050	7.36 (0.56–95.92)	0.127
Secondary	10.06 (1.23–82.33)	0.031	10.99 (0.79–152.17)	0.074
Tertiary	1 (ref)		1	
Area residence				
Rural	1			
Urban	0.94 (0.37–2.37)	0.892		
Poverty line				
Above	1		1	
Below	1.96 (0.77–4.95)	0.157	7.15 (1.39–36.67)	0.018
Substance use				
No	1			
Yes	0.64 (0.20–2.06)	0.458		
Distance from the hospital	1.02 (0.99–1.03)	0.108	1.01 (0.98–1.04)	0.521
Presence of a comorbidity				
No	1		1	
Ye	1.78 (0.61–5.20)	0.293	2.05 (0.40–10.64)	0.391
Insight for psychosis				
Good	1			
Poor	0.85 (0.29–2.51)	0.772		
Attitude towards typical antipsychotic				
Negative	1			
Positive	1.40 (0.48–4.11)	0.538		
Duration of treatment (in years)	0.97 (0.91–1.04)	0.456		
Satisfaction with medication	1.23 (0.77–197)	0.379		
Burdened by the number of pills				
No	1			
Yes	1.08 (0.42–2.81)	0.868		
Number of pills	0.79 (0.60–1.04)	0.091	0.85 (0.52–1.38)	0.511
Frequency of daily drug taking				
Once	1		1	
Twice	0.56 (0.21–1.52)	0.257	0.43 (0.08–2.18)	0.308
Three times	0.27 (0.03–2.28)	0.232	0.36 (0.02–7.32)	0.507
Four or more times	3.85 (0.22–65.71)	0.352	4.18 (0.08–228.96)	0.483
Frequency of drug stoke out at the facility for patients' medications				
Never	1			
Rarely	2.12 (0.20–21.88)	0.529		
Sometimes	1.64 (0.19–14.37)	0.657		
Often	1.42 (0.13–15.64)	0.774		
Always	2.77 (0.26–29.04)	0.396		
Level of social support from family and friends	0.87 (0.56–1.37)	0.559		
Satisfaction with health workers' explanations of drug use and side effects	0.91 (0.65–1.28)	0.595		
Stop medications due to religious or cultural beliefs				
No	1			
Yes	1.27 (0.49–3.31)	0.620		
Loss of energy or drive				
No	1		1	
Yes	0.22 (0.09–0.59)	0.002	0.15 (0.03–0.81)	0.028
Feeling unmotivated				
No	1		1	
Yes	0.41 (0.16–1.09)	0.073	2.18 (0.35–13.47)	0.401
Day time sedation				
No	1		1	
Yes	0.59 (0.24–1.49)	0.266	1.59 (0.36–6.99)	0.535
Too much sleep				
No	1			
Yes	0.84 (0.33–2.13)	0.715		
Muscle too stiff				
No	1			
Yes	1.42 (0.52–3.83)	0.492		
Shaking				
No	1			
Yes	0.61 (0.22–1.68)	0.339		
Jittery				
No	1			
Yes	0.63 (0.21–1.84)	0.397		
Akathisia				
No	1		1	
Yes	0.48 (0.13–1.74)	0.264	0.68 (0.12–3.94)	0.671
Trouble waking up				
No	1			
Yes	0.95 (0.38–2.36)	0.909		
Vision problems				
No	1		1	
Yes	0.49 0.16–1.57)	0.233	0.42 (0.08–2.06)	0.282
Dry mouth				
No	1			
Yes	0.88 (0.33–2.35)	0.807		
Drooling of saliva				
No	1			
Yes	0.89 (0.24–3.34)	0.865		
Memory problems				
No	1			
Yes	0.94 (0.38–2.36)	0.901		
Constipation				
No	1		1	
Yes	1.73 (0.66–4.57)	0.265	2.45 (0.59–10.13)	0.217
Weight changes				
Decrease	0.29 (0.06–1.54)	0.148	1.34 (0.18–9.85)	0.772
Increase	0.60 (0.22–1.64)	0.318	6.66 (0.73–60.51)	0.092
No change	1		1	
Sexual arousal problems				
No	1			
Yes	1.06 (0.42–2.65)	0.901		
Menstrual changes				
No	1		1	
Yes	2.27 (0.77–6.67)	0.135	1.22 (0.26–5.60)	0.798
Total number of side effects experienced	0.89 (0.76–1.04)	0.138		

## Data Availability

The datasets used in the analysis during the current study are available at doi:10.6084/m9.figshare.19653609.
